# Evidence-based framework for organisational best practice recommendations in healthcare governance

**DOI:** 10.3389/frhs.2026.1803273

**Published:** 2026-04-17

**Authors:** Davide Di Fusco, Serena Lavorgna, Barbara Rossi, Matteo Marconi, Claudia Marchetta, Gabriella Facchinetti, Marco Bressi, Filippo Lauria, Romano Arcieri, Velia Bruno

**Affiliations:** National Centre for Clinical Governance and Excellence in Health Care, Italian National Institute of Health, Rome, Italy

**Keywords:** clinical best practice recommendations, clinical governance, evidence-based medicine, guideline, healthcare organization

## Abstract

Clinical governance requires the integration of evidence not only on clinical interventions but also on organisational processes that shape health services delivery and directly influence quality, efficiency, and patient safety. Despite the widespread availability of evidence-based clinical guidelines, healthcare systems often lack standardised methods to translate evidence into effective organisational models. To address this gap, the Italian National Centre for Clinical Governance (CNCG) at the Italian National Institute of Health (ISS) developed a structured methodological framework for Organisational Clinical Best Practice Recommendations (O-BPCA), aimed at strengthening evidence-based governance and organisational performance within healthcare services. The O-BPCA framework was developed through a structured, multi-step process encompassing governance principles, methodological standards, and operational phases, culminating in institutional validation through external review, public consultation, and formal approval by the ISS. The methodological manual defines transparent procedures for evidence synthesis, consensus building, and the formulation of measurable organisational recommendations applicable across health service settings. The framework has been operationalised within the CNCG through the identification of two priority macro-areas for organisational innovation, surgical and territorial care, and the establishment of dedicated Thematic Operational Groups (GOTs) responsible for developing and validating O-BPCA recommendations in selected priority domains. Early implementation has highlighted the framework's feasibility, scalability, and potential to support coordinated organisational improvement within the Italian National Health Service. The O-BPCA framework represents an innovative policy and methodological tool for healthcare governance, bridging clinical evidence and organisational implementation within health services. By promoting standardisation, transparency, and accountability in organisational decision-making, the framework supports sustainable improvements in service delivery and performance. Its structured and replicable approach makes it transferable beyond the Italian National Health Service and adaptable to other health systems seeking to strengthen evidence-based organisational governance.

## Introduction

Delivering high-quality, efficient, and safe healthcare requires an integrated approach that encompasses both clinical excellence and the governance of organisational structures and processes. The concept of “Clinical governance”, introduced in the late 1990s, was conceived as a framework through which healthcare organisations could be held accountable for continuously improving service quality within systems that uphold high standards of care (1). While considerable progress has been made in guideline development, auditing and patient safety, the organisational dimension of healthcare, how services are structured, managed and coordinated, remains comparatively underdeveloped and inconsistently addressed across health systems.

In Italy, this gap has been formally recognised within the national legal and institutional framework. Law N. 24/2017 (“Gelli-Bianco Law”) established the principle that healthcare quality and safety depend on both professional competence and organisational reliability (2). The law assigned to the Italian National Institute of Health (ISS) the responsibility for coordinating the National Guidelines System (SNLG), the national guideline system, to ensure methodological rigour, transparency and accessibility of clinical and organisational recommendations. Importantly, the Gelli-Bianco Law also introduced a legal mechanism for the protection of healthcare professionals, linking professional accountability to the adoption of nationally validated guidelines and Best Practice Recommendations (BPCA). In this context, adherence to evidence-based and institutionally endorsed recommendations represents not only a standard for quality and safety but also a safeguard for professionals operating within the Italian National Health Service. Within this regulatory and institutional framework, the National Centre for Clinical Governance and Excellence in Health Care (CNCG) of the ISS was entrusted with developing methodologies and tools to strengthen evidence-based governance and to promote the systematic production of BPCA.

Despite the availability of evidence-based clinical guidelines and BPCA, healthcare organisations often lack structured, standardised methods to translate this evidence into effective organisational models. Variability in organisational models and health system performance can compromise quality, safety, and equity of care (3, 4).

International institutions, including the World Health Organization (WHO), the Organisation for Economic Co-operation and Development (OECD), and the European Network for Health Technology Assessment (EUnetHTA), have consistently emphasised the need for methodological frameworks that integrate clinical evidence with organisational implementation (5–7), ensuring that quality improvement and safety initiatives are embedded within system-level governance. However, existing international frameworks, such as those developed by the WHO, National Institute for Health and Care Excellence (NICE) and Agency for Healthcare Research and Quality (AHRQ), mainly provide conceptual or guidance-level approaches without a unified, standardised methodology for developing organisational recommendations applicable at national or regional scales. To address this gap, ISS developed a methodological framework for Organisational Clinical Best Practice Recommendations (O-BPCA). This framework provides a reproducible, evidence-based and consensus-driven process for producing organisational recommendations that complement existing clinical guidelines. Reproducibility refers to the use of a structured and standardised methodological pathway, including evidence synthesis and appraisal, multidisciplinary expert involvement, formal consensus procedures, external review, public consultation, institutional validation, and transparent reporting. It integrates international methodological standards within a nationally coordinated governance model (8–10), based on the institutional oversight and methodological coordination ensured by the ISS within the Italian National Guidelines System (SNLG), in accordance with Law 24/2017.

Table 1 summarises these international models and highlights the distinctive contribution of the ISS O-BPCA framework, which implements these principles through a structured, transparent and institutionally validated process for developing organisational best practice recommendations.

**Table 1:** Governance structure and distinctive features of international frameworks for evidence-based organisational practice compared to O-BPCA model.

Framework/institution	Primary focus	Methodological core	Governance and implementation	Distinctive features compared to O-BPCA
WHO Quality of Care Framework/IPCHS	System-level improvement of care quality, safety, and coordination	Conceptual model integrating structure, process, and outcome dimensions	Global reference adaptable to national systems; no prescriptive method for developing recommendations	Provides guiding principles for quality and system design; O-BPCA operationalises these into a structured national methodology

NICE Service Delivery and Organisation Guidelines	Evidence-based improvement of service organisation and delivery	Systematic evidence synthesis and GRADE-based consensus process	Developed by topic-specific expert groups under NICE oversight; implemented within NHS Trusts	Focused on local service redesign; O-BPCA extends to national governance with institutional validation and measurable indicators

AHRQ Improving Organisation of Care Framework	Translating evidence into system improvement and patient safety	Evidence-to-implementation approach using frameworks like CFIR and Learning Health Systems	Decentralised adoption by local and federal agencies	Promotes implementation and innovation diffusion without national standardisation. O-BPCA introduces unified governance and methodological validation

O-BPCA Italian National Institute of Health	Standardisation of organisational clinical practices within the Italian NHS	Structured multi-step process: evidence synthesis, consensus, validation, publication	Centralised institutional governance ensuring transparency and quality control	First national model integrating methodological rigour, institutional validation and policy transferability


Within this framework, the O-BPCA methodology underlines that organisational best practice recommendations should explicitly define the intended goals, such as improving access, timeliness, appropriateness and continuity of care, without prescribing specific structural or technological solutions. The recommendations are designed to be implemented within existing resources, promoting optimization and integration rather than expansion. This approach encourages the re-organization of available assets through network-based models, shared care pathways, and inter-institutional collaboration, ensuring economic sustainability and operational feasibility (11, 12).

Despite the growing emphasis on evidence-based clinical practice, structured and reproducible methodological frameworks specifically designed to develop organisational recommendations, and formally integrated within national healthcare governance systems, remain limited in the current literature.

In this context, the present study describes the implementation of the O-BPCA framework by the CNCG of the ISS, outlining its governance architecture and operational structure as a structured and transferable methodological model for integrating evidence, consensus, and governance processes in the development of organisational best practice recommendations.

## Materials and methods

### Governance structure

The development of O-BPCA follows a structured, evidence-based and consensus-driven methodology defined by the CNCG of the ISS. The process ensures scientific rigour, methodological transparency and multidisciplinary participation, in accordance with ISS Presidential Decree N. 37/2025 and the national framework established by Law 24/2017.

Three inter-related entities coordinate the entire development process:

1. *Thematic Operational Group (GOT)*
  Thematic Operational Groups are working units established for each priority area (e.g., surgery, vascular access, chronic diseases, frailty, and disability). Each group defines the organisational topic, identifies the relevant scientific and professional societies to be involved, and formally appoints the group of scientific societies responsible for developing the O-BPCA document. Within this multidisciplinary group, to support coordination and ensure methodological and organisational alignment throughout the process, the GOT designates a lead society or association with recognised scientific and clinical expertise in the thematic field. In addition, to guarantee full supervision and continuity between institutional and professional partners two representatives are nominated, one internal to the ISS and one external. Each group may also identify within its membership one or more individuals with highly specialised technical or organisational expertise relevant to the topic. These professionals may be engaged as external consultants to provide targeted input and to strengthen the scientific and contextual quality of the recommendations. Upon completion of the drafting process, the GOT receives and reviews the final O-BPCA document, verifying its coherence with the strategic vision and thematic scope initially defined. The validated document is then formally transmitted to the CNCG for methodological quality assessment and institutional approval.
2. *Multisociety Group*
  This group is composed of representatives from scientific and professional societies with recognised expertise and direct involvement in the topic addressed by the O-BPCA. The multisociety group is directly responsible for coordinating the drafting of the document, including the organisation of activities, communication among participating societies and overall logistical management.
  Within of the group, one lead society or association is designated to facilitate coordination and ensure continuity of communication across partners. The lead society acts as the main operational interface, providing logistical support and submitting the final O-BPCA document to the GOT for review and validation. This role, however, does not confer any hierarchical authority: all participating societies collaborate on an equal basis throughout the development process, contributing equally to the scientific and organisational content of the recommendations.
  The multisociety group appoints up to two coordinators to supervise the development process and ensure methodological alignment. Their specific roles and responsibilities are detailed in the following section.
3. *Development Group*
  The development group constitutes the operational core of the O-BPCA development process. Appointed by the multisociety group and established under the supervision of the GOT, it brings together professionals with complementary expertise to ensure a scientifically rigorous, transparent, and multidisciplinary approach.
  The development group is responsible for the methodological, technical, and organisational execution of all activities related to the formulation of the O-BPCA, from defining the scope and conducting literature searches to drafting statements, supporting rationales, and measurable indicators. The composition, roles, and responsibilities of the development group are summarized in Table 2.
  Briefly, the coordinator leads and supervises the overall development process, ensuring coherence and adherence to methodological standards. The methodologist guarantees scientific integrity and methodological rigour across all phases. Literature search specialists are responsible for systematic evidence retrieval and documentation, while the expert panel, a multidisciplinary body, integrates clinical, organisational, ethical, and patient perspectives. External reviewers provide independent evaluation of scientific quality and applicability, and external consultants offer specialised expertise when required. The secretariat ensures administrative support, coordination, and documentation throughout the process.

**Table 2:** Composition, roles, and responsibilities of members within the development group.

Role within the development group	Key responsibilities
Coordinator	Oversees and manages the O-BPCA development process; ensures methodological compliance and timelines; defines objectives with the GOT; coordinates the group and workflow; supervises consensus procedures and documentation; manages communication and conflicts of interest; does not participate in voting.
Methodologist	Ensures scientific integrity and methodological rigour; defines methodological strategy and evidence review; supervises literature search; designs and manages consensus processes (e.g., NGT, Delphi); ensures transparency and traceability; supports training and documentation; does not participate in voting.
Literature search specialists	Conduct systematic and reproducible literature searches; apply inclusion/exclusion criteria; document search strategies; support screening, data extraction, and evidence synthesis; do not participate in voting.
Expert panel	Multidisciplinary and multiprofessional group, representative across healthcare settings and regions; members are selected based on predefined criteria, including recognised expertise, and where appropriate, years of experience, scientific or academic contributions and relevant clinical, organisational, or methodological involvement; contributes to scope definition, statement formulation, and consensus; integrates clinical, organisational, ethical, and patient perspectives; ensures applicability and declares conflicts of interest.
External reviewers	Provide independent evaluation of scientific quality, methodological soundness, and applicability; at least two reviewers involved; feedback discussed before finalization.
External consultants	Provide specialized expertise when required; contribute advisory input; all contributions documented and assessed; do not participate in voting.
Secretariat	Provides administrative, logistical, and communication support; organizes meetings; manages documentation and version control; ensures coordination, traceability, and compliance with institutional standards.

Each member of the development group operates within clearly defined roles and responsibilities to ensure that the O-BPCA is developed according to the methodological standards and quality criteria established by the ISS.

### Development of the framework

**Figure 1:**
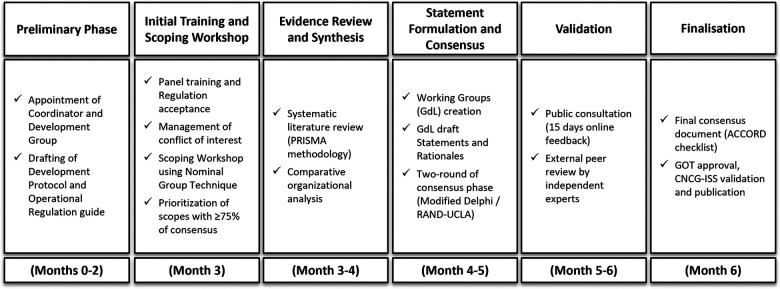
Development process of O-BPCA: Methodological phases. The figure summarizes the phases of the O-BPCA development pathway and their relative timeline: governance setup, methodological training and scoping, systematic evidence review, statement formulation with a structured consensus process, and validation and finalisation. The final phase is represented by two sequential steps (validation and finalisation), displayed as separate boxes in the flowchart.

The development of O-BPCA follows a structured, multi-phase process designed to ensure methodological rigour, transparency, and reproducibility. This section describes the methodological development and structure of the O-BPCA framework. The framework is designed to be reproducible across different organisational contexts through predefined and standardised methodological steps, including evidence synthesis and appraisal, structured consensus procedures, and formal processes for validation, reporting, and institutional approval. Each phase includes defined activities, responsibilities and timelines, as showed in Figure 1. The process consists of five different phases:

1. Preliminary phase;
2. Initial training and scoping workshop;
3. The evidence review and synthesis;
4. Statement formulation and consensus;
5. Validation and finalisation.

#### The preliminary phase

The preliminary phase establishes the organisational and procedural foundations for O-BPCA development. During this stage, the coordinator and other members of the development group are appointed, roles and responsibilities are defined, and the modalities for meetings, documentation management, and communication are formalised. This phase should be completed within two months and includes the preparation of two essential documents: the development protocol and the operational regulation guide.

The development protocol provides a methodological roadmap for the entire O-BPCA process. It includes the objectives of the development group, the thematic priorities and potential topics, the methodological approach adopted, a detailed timeline for each phase, the responsibilities division among members and dissemination strategies to support the communication and implementation of the recommendations.

The operational regulation guide defines the procedural and governance rules that ensure transparency and consistency throughout the process. It details the roles and responsibilities of all members, the communication methods and meeting procedures, the criteria for voting, decision-making and minimum consensus thresholds (≥75% of agreement), the management of confidentiality and conflicts of interest and finally the rules for drafting, approving, and publishing the final document.

To note, both documents must be completed prior to the first plenary meeting (scoping workshop) to guarantee a clear and structured decision-making framework.

#### Initial training and scoping workshop

The first plenary meeting marks the formal initiation of the O-BPCA development process and serves two main purposes: (1) to provide introductory training for all members of the expert panel and (2) to conduct a scoping workshop to identify and prioritise the main organisational themes (scopes) to be developed. This meeting must take place within three months from the beginning of the process.

During the training session, the methodologist, supported by the coordinator, conducts a structured orientation to ensure that all participants share a consistent understanding of the methodological framework and operational procedures adopted for O-BPCA development. The session covers the key methodological principles and minimum quality requirements, the specific roles and responsibilities of all members, the process for scope prioritisation and statement formulation, the application of reference standards, and the management of conflicts of interest. Participants are encouraged to express any additional training needs to enhance their active engagement in the process. At the end of the session, all members formally sign the operational regulation, thereby confirming their commitment to the agreed procedural rules, confidentiality standards, and collaborative principles.

The scoping workshop aims to identify and prioritise the most relevant organisational questions within the assigned thematic area. Moderated jointly by the coordinator and the methodologist, the workshop employs structured brainstorming and guided discussion techniques to foster inclusive participation and balanced contribution from all panel members. The use of the nominal group technique is recommended to ensure systematic idea generation, equitable participation, and the mitigation of dominance bias (13). Each participant individually proposes potential scopes, which are subsequently discussed, clarified, and collectively refined.

Following discussion, an anonymous prioritisation process is conducted using a 9-point Likert-type scale (1–3 = non-priority; 4–6 = uncertain; 7–9 = priority). Consensus is considered achieved when at least 75% of experts (excluding the methodologist and literature search specialists) score within the same interval (14). Scopes reaching this threshold are selected for further development and guide the next phases of the O-BPCA process.

Once the scoping workshop is completed, the development protocol, previously drafted by the coordinator and the methodologist, is updated and formally adopted as the official methodological reference guiding all subsequent stages of the O-BPCA development process. The protocol consolidates the objectives, timelines, assigned responsibilities, and methodological standards defined during the workshop, ensuring coherence, transparency, and reproducibility throughout the process.

#### Evidence review and synthesis

This phase represents the scientific core of the O-BPCA process. It involves a systematic and structured review of the available evidence to ensure that each statement and rationale is supported by current and robust knowledge.

The literature search specialists, under the supervision of the methodologist, perform comprehensive literature searches primarily in PubMed (https://pubmed.ncbi.nlm.nih.gov), complemented by at least one additional specialised database such as Embase (https://www.embase.com) for broader biomedical and organisational research coverage. The review process follows PRISMA (Preferred Reporting Items for Systematic Reviews and Meta-Analyses) guidelines or equivalent validated checklists to ensure transparency and reproducibility (13). Depending on the scope, the evidence synthesis may take the form of a systematic review, rapid review, or qualitative meta-synthesis (15). Eligible sources include original research articles, systematic or narrative reviews, meta-analyses, position papers and national or international guidelines. Qualitative sources (e.g., focus groups, interviews, surveys, and participatory research) are integrated when appropriate to provide contextual understanding.

By combining quantitative and qualitative methods, the O-BPCA process promotes a value-based healthcare perspective, linking organisational interventions to measurable outcomes through PROMs (Patient-Reported Outcome Measures) and PREMs (Patient-Reported Experience Measures).

This phase should be completed within four months from the start of the Preliminary Phase.

#### Comparative analysis

In addition to the literature review, a comparative analysis of the current organisation of services within the thematic area under study may be conducted. This analysis aims to examine structural and functional variations across healthcare settings and to assess the performance associated with different organisational models. Such information is essential to support an evidence-based and context-sensitive evaluation, ensuring that the proposed recommendations are grounded in real-world practices and operational feasibility.

The comparative analysis contributes to identifying strengths, gaps, and variability in existing service models, taking into account contextual factors such as institutional size, resource availability, and organisational capacity. These insights are crucial for formulating statements and defining minimum quality criteria that are both evidence-informed and realistically applicable across diverse healthcare contexts. This process also supports the development of sustainable and adaptable organisational models, promoting equity and consistency in care delivery regardless of regional or structural disparities. Where appropriate, the development group may gather complementary data or consult existing analytical frameworks to enrich the evaluation and strengthen the empirical basis of the O-BPCA recommendations.

#### Statement formulation and consensus process

Following evidence synthesis and scope prioritisation, the development group proceeds to the operational phase of statement formulation. Each statement represents a structured, evidence-informed assertion expressing the collective judgement of the expert panel. Statements must be formulated in clear, neutral, and actionable terms (e.g., “the Panel considers that…”, “the Panel recommends that…”), supported by a transparent rationale grounded in the most up-to-date scientific and organisational evidence identified through the systematic review and comparative analysis.

To manage this phase effectively, the expert panel may be organised into multidisciplinary working groups, each responsible for drafting statements and supporting rationales within specific thematic sub-areas. Where appropriate, external consultants may be involved to contribute specialised expertise or experiential insights, enriching the formulation process with real-world perspectives. The coordinator and the methodologist review all draft statements to ensure scientific integrity, linguistic clarity, and alignment with the methodological standards defined in the development protocol. Each working group is also expected to identify minimum quality requirements and monitoring indicators, both process indicators, to track implementation and operational performance, and outcome indicators, to measure the effectiveness and impact of the proposed organisational practices. This ensures that every recommendation integrates a measurable framework for continuous evaluation, consistent with the principles of quality improvement, accountability, and patient safety.

Once the draft statements and rationales are finalised, the consensus process is activated to validate them through a structured, iterative, and transparent procedure. The process is inspired by internationally recognised consensus methodologies, the Nominal Group Technique (NGT), the Modified Delphi Method, and the RAND/UCLA Appropriateness Method, ensuring inclusiveness, methodological rigour and reproducibility across successive rounds of evaluation (13–17). These procedures incorporate predefined agreement thresholds and structured voting metrics, providing a transparent and reproducible basis for consensus evaluation (18).

Under the supervision of the coordinator and the methodologist, the panel evaluates each statement through an anonymous voting procedure using a 5-point Likert-type scale (1 = strong disagreement; 5 = strong agreement). Scores of 3 indicate neutrality or uncertainty; while they do not contribute to agreement or disagreement, their prevalence may lower the overall level of consensus and prompt clarification or revision before a second voting round. Consensus is considered achieved when at least 75% of participants (excluding the Coordinator, Methodologist, and literature search specialists) assign a score within the same agreement range (1–2 = disagreement; 4–5 = agreement) (18).

The consensus process consists of two main rounds:

1. First round—Anonymous electronic voting. Participants may provide open comments to justify their ratings or suggest modifications to statements and rationales.
2. Second round—Conducted only for statements that did not meet the consensus threshold or were revised after feedback. This round focuses exclusively on the revised items; open commentary is not permitted to ensure convergence and stability of agreement.

All voting results are summarised in a comprehensive consensus report, documenting distributions, agreement levels, and the rationale for any modifications between rounds (18). These procedures define a structured voting process based on predefined agreement criteria and quantitative thresholds, ensuring transparency and reproducibility of the consensus process.

This multi-step strategy, combining quantitative scoring and qualitative feedback, ensures that each statement reflects both the strength of available evidence and the collective, multidisciplinary agreement of experts. The phase should be completed within five months from the start of the preliminary phase, ensuring methodological consistency and adherence to the established timeline.

#### Validation and finalisation

The validation phase ensures both the scientific robustness and institutional transparency of the O-BPCA development process through structured public consultation and external review. This phase must be completed within six months from the initiation of the preliminary phase.

#### Public consultation

The draft O-BPCA document is made publicly available on the multisociety group online platform for a 15-day consultation period, during which stakeholders may provide written comments and rate each statement using a 1–5 scale.

To assess the relevance and appropriateness of proposed revisions, all feedback is anonymised and systematically reviewed by the coordinator and methodologist in collaboration with the expert panel. Accepted changes are integrated into the revised draft, and a full anonymised record of all comments, together with corresponding responses and justifications, is annexed to the final report.

This open consultation phase reinforces transparency, promotes stakeholder engagement, and supports alignment between professional, institutional and patient perspectives.

#### External review

To ensure methodological accuracy and scientific validity, at least two independent external reviewers are appointed by the coordinator based on their recognised expertise in the relevant clinical-organisational domain.

Reviewers evaluate the draft O-BPCA for coherence with methodological standards, internal consistency, and practical applicability within healthcare settings. Their feedback is summarised in written reports, which are subsequently discussed with the expert panel before the document's finalisation. This independent evaluation represents a key safeguard for the credibility and generalisability of the final recommendations (13–19).

#### Final document and institutional validation

Following the conclusion of both review steps, the coordinator, with support from the secretariat, arranges the final consensus document in accordance with the ACCORD checklist, ensuring compliance with recognised reporting standards (10, 20).

The final version integrates detailed rationales for each statement, the methodological description, and anonymised records of all comments and reviewer reports, thereby ensuring full traceability of the decision-making process. The completed O-BPCA document is then submitted to the GOT for formal approval. Once endorsed, it is formally transmitted to the CNCG of the ISS for methodological quality assessment by institutional methodologists. If the document meets the required methodological and quality standards, it is validated and officially published on the institutional website of the ISS, as part of the SNLG, thereby ensuring public accessibility and national recognition. This final step ensures national consistency, transparency and official recognition within the SNLG framework.

## Results

This section presents the implementation of the O-BPCA framework within the CNCG, illustrating its application in real-world organisational contexts. Preliminary findings highlight its strategic potential as a policy instrument to strengthen evidence-based governance within the Italian National Health Service. Developed under the coordination of the CNCG, the framework has demonstrated its capacity to link the generation of scientific evidence with the continuous improvement of organisational quality, safety and efficiency.

Through a nationally coordinated process, two thematic macro-areas were identified as priorities for organisational innovation: “Surgical Appropriateness and Organisational Quality” and “Territorial Appropriateness and Organisational Care Models”. Within these domains GOTs were formally established with ISS agreement to develop and validate O-BPCA recommendations.

The GOTs of surgical macro-area are currently developing organisational recommendations addressing key domains of healthcare delivery, including vascular surgery emergencies (e.g., acute aortic syndrome and abdominal aortic rupture), anaesthesia and critical care innovation, hospital management of adult oncology patients, reconstructive plastic surgery and aesthetic medicine, with a focus on the appropriate and safe use of prosthetic and injectable materials, major ambulatory surgery, vascular access in nephrology, with emphasis on organisational requirements for patients with advanced chronic kidney disease, preoperative assessment and optimization in ASA I–II patients and operating room resource efficiency.

In the territorial macro-area, the GOTs are formulating recommendations on home-based vascular access management, vascular access in nephrology, transitional care pathways and the multidisciplinary management of chronic conditions such as psoriasis and osteoporosis. GOTs established within the O-BPCA framework and their corresponding recommendations are reported in Table 3.

**Table 3:** Overview of GOTs and corresponding O-BPCA recommendations in surgical (“surgical appropriateness and organisational quality”) and territorial (“territorial appropriateness and organisational care models”) macro-area.

Macro-area	GOT	O-BPCA
Surgical appropriateness and Organisational quality	Vascular surgery emergencies	Acute aortic syndrome and abdominal aortic rupture
	Anaesthesia, intensive care and critical care area	Technological innovation in the perioperative setting
		Hospital management of the adult oncology patient
	Reconstructive plastic surgery and aesthetic medicine	Management of pre-, intra- and post-operative phases in rhinoplasty
		Management of pre-, intra- and post-operative phases in liposuction and lipofilling
		Clinical-Organisational appropriateness in the use of prosthetic materials for aesthetic purposes (Mammary and Gluteal Implants)
		Clinical-Organisational appropriateness in the practice of dermal fillers
	Major ambulatory surgery	Clinical, assistential and Organisational appropriateness in major ambulatory surgery
	Vascular access in nephrology	Organisational requirements for the management of vascular access in advanced chronic kidney disease patients
	Preoperative preparation (ASA I–II patients)	Preoperative preparation for ASA I-II patients in ambulatory and day surgery settings
	Operating room resource optimisation	Optimisation and efficiency of operating room resources
Territorial appropriateness and Organisational care models	Home-based vascular access	Implantation and management of vascular access devices in home care
	Transitional care	Guarantee criteria, methodology and organisational appropriateness to transitional care access
	Psoriasis	Multidisciplinary management of adult patients with psoriasis
	Osteoporosis	Multidisciplinary management of adult patients with osteoporosis

Early implementation in pilot regional contexts has demonstrated the framework's feasibility and scalability, consolidating its role as a national policy instrument for evidence-based organisational innovation. Within this structure, each GOT functions as a multidisciplinary and cross-institutional working group, translating the O-BPCA methodology into actionable organisational recommendations that promote standardization, transparency, and accountability across the Italian National Health Service.

## Discussion

This study presents the O-BPCA framework as the first national methodological manual specifically designed for the development of organisational clinical best practice recommendations. The framework provides a transparent, evidence-informed, and standardised process that integrates institutional, scientific, and professional stakeholders within a shared governance architecture, addressing a well-recognised gap between clinical evidence production and organisational implementation in healthcare systems. Consistent with the principles established by Italian Law No. 24/2017, the O-BPCA framework reinforces the interdependence between professional competence and organisational reliability, recognising both as essential determinants of quality and patient safety. By embedding organisational recommendations within a formally governed and institutionally validated process, the framework strengthens accountability while supporting consistent decision-making across healthcare services. A distinctive feature of the framework is its principle of economic rationality, which promotes optimization and integration of existing resources rather than the introduction of new structural or technological assets. This approach supports organisational innovation that is both feasible and sustainable, encouraging re-organization through network-based models, shared care pathways, and inter-institutional collaboration.

In this perspective, the O-BPCA framework can be interpreted not only as a methodological tool but also as a policy instrument to support evidence-based organisational governance at system level. Its early implementation within the CNCG of the ISS demonstrates how structured methodologies can be translated into coordinated national initiatives, enabling the identification of priority domains and the development of actionable organisational recommendations. While initial applications have demonstrated feasibility and organisational applicability, comprehensive quantitative evaluation data are not yet available. Future phases will focus on the systematic assessment of the framework's impact through predefined indicators, including consensus dynamics, implementation timelines, and organisational performance measures. The framework also supports organisational adoption and change management by promoting stakeholder engagement, multidisciplinary collaboration, and the integration of recommendations within existing care pathways and governance structures. This approach facilitates the translation of evidence into practice while ensuring alignment with local organisational contexts.

Beyond its national scope, the O-BPCA framework offers a replicable international model for evidence-based organisational governance. From a health policy perspective, this approach is relevant for the international community, as it provides a methodological reference to support the standardisation, implementation, and evaluation of organisational practices within healthcare systems. While the framework is designed to be transferable, its application may require adaptation to different healthcare system structures and governance models. Future developments will focus on scaling the framework at national level and exploring its transferability to other healthcare systems. This includes the development of structured evaluation strategies and performance indicators to assess its impact on organisational quality, safety, and efficiency, as well as its adaptability across different governance contexts.

This study has some limitations. The development and implementation of the O-BPCA framework require a structured and resource-intensive process, which may be time-consuming and dependent on strong institutional coordination. In addition, organisational and administrative barriers may affect the adoption and scalability of the framework across different healthcare settings. Furthermore, although the framework has entered an early implementation phase, comprehensive quantitative evaluation data are not yet available. Future studies will be needed to assess its impact on organisational performance, quality of care, and patient outcomes.

Overall, by bridging the gap between clinical evidence and healthcare system implementation, the O-BPCA framework provides a structured, scalable and sustainable approach to strengthening quality, safety and accountability within complex health systems.

## Data Availability

The raw data supporting the conclusions of this article will be made available by the authors, without undue reservation.
